# Perspectives on Functional Red Mold Rice: Functional Ingredients, Production, and Application

**DOI:** 10.3389/fmicb.2020.606959

**Published:** 2020-11-25

**Authors:** Feng Yanli, Yu Xiang

**Affiliations:** ^1^Hubei Key Laboratory of Edible Wild Plants Conservation and Utilization, Hubei Normal University, Huangshi, China; ^2^Hubei Engineering Research Center of Typical Wild Vegetables Breeding and Comprehensive Utilization Technology, Hubei Normal University, Huangshi, China; ^3^National Demonstration Center for Experimental Biology Education, Hubei Normal University, Huangshi, China; ^4^College of Life Sciences, Hubei Normal University, Huangshi, China

**Keywords:** functional red mold rice, monacolins, functional ingredients, production, application, *Monascus* spp.

## Abstract

Monacolin K (MK) is a secondary metabolite of the *Monascus* species that can inhibit cholesterol synthesis. Functional red mold rice (FRMR) is the fermentation product of *Monascus* spp., which is rich in MK. FRMR is usually employed to regulate serum cholesterol, especially for hypercholesterolemic patients who refuse statins or face statin intolerance. The present perspective summarized the bioactive components of FRMR and their functions. Subsequently, efficient strategies for FRMR production, future challenges of FRMR application, and possible directions were proposed. This perspective helps to understand the present situation and developmental prospects of FRMR.

## Introduction

Red mold rice (RMR), also called red koji or red yeast rice is the fermentation product of *Monascus* spp. (Farkouh and Baumgärtel, 2019). It is widely used as a colorant, supplement, and starters in the food industry in Asian countries. RMR contains multiple beneficial metabolites, such as *Monascus* pigments, monacolin K (MK), and γ-aminobutyric acid. RMR also contains some enzymes, for instance, protease and amylase (De Backer, 2017; Chen et al., 2019; Jiang et al., 2019). However, a mycotoxin-citrinin produced by *Monascus* spp. can induce health risks (He et al., 2020b). Nowadays, RMR has three main product types on the market depending on its application, as follows: coloring RMR, brewing RMR, and functional red mold rice (FRMR). Coloring RMR is the RMR with a color value higher than 1,000 U/g according to the National Food Safety Standard of China (GB 1886.19–2015). Brewing RMR is the RMR that possesses strong saccharifying power and esterifying power, which is used as a fermentation starter in the food industry based on the Light Industry Standard of the People’s Republic of China (QB/T 5188–2017). FRMR is the RMR with a natural MK of more than 0.4% according to the Light Industry Standard of the People’s Republic of China (QB/T 2847–2007). MK is chemically identical to lovastatin, which is a lipid-lowering drug and shows evident effects on inhibiting 3-hydroxy-3-methylglutaryl-CoA (HMG-CoA) reductase that catalyzes the rate-limiting step of cholesterol biosynthesis (De Backer, 2017; Bruno et al., 2018). Therefore, FRMR is a commonly consumed food supplement by hypercholesterolemia patients, especially for statin-intolerant community (Mazzanti et al., 2017; Xiong et al., 2019).

However, some issues related to FRMR should be taken into consideration. Firstly, MK has a large number of analogs with different lipid-lowering effects and complex conversion relationships (Kimura et al., 1990; Li et al., 2017; Beltrán et al., 2019). For instance, 84 monacolins (MLs) have been monitored in RMR sample (Li et al., 2017). FRMR available from market contains different contents of MK and different MLs in each FRMR sample (Dujovne, 2017). Therefore, it is inappropriate to define the functions of FRMR by merely depending on its MK content. Secondly, MK possesses specific active forms and has multiple functions. MK is an inactive lactone, which needs to be converted into its active β-hydroxy acid form (MKA) for the lipid-lowering activity (Yang and Hwang, 2006). Moreover, some other functions of MLs have also been reported, including promoting bone formation, being an antioxidant, and suppressing cancer cell proliferation (Wong and Rabie, 2008; Kurokawa et al., 2017; Nagabhishek and Madankumar, 2019; del Gaudio et al., 2020). Thirdly, a great number of metabolites besides MLs are available from FRMR. The functions of the beneficial metabolites such as *Monascus* pigments and γ-aminobutyric acid should be understood and evaluated, while using FRMR as a food supplement or as an alternative drug to the chemical statins. Meanwhile, the toxic metabolite produced by some *Monascus* strains, citrinin, should not only be studied but should also be carefully controlled (Farkouh and Baumgärtel, 2019). Furthermore, the side effects of MLs such as myopathies and liver injury need to be evaluated (Mazzanti et al., 2017). For the efficient application of FRMR, MK contents of FRMR should be standardized, and the functions and safety of FRMR need to be evaluated.

To obtain sufficient MK in FRMR, parameters for FRMR production in solid- and liquid-state fermentation such as initial moisture, pH, and nitrogen source have been optimized (Hp, 2012; Feng et al., 2014; Lin et al., 2017; Huang et al., 2018). Moreover, the screening of *Monascus* strains with high-MK production has also been carried out (Suh et al., 2007; Wang et al., 2011). Novel substrates have been utilized for FRMR production and enriching its product types, such as *Dioscorea*, *Finger millet*, and *Saccharina japonica* (Lee et al., 2007; Venkateswaran and Vijayalakshmi, 2010; Suraiya et al., 2018). All these strategies head for high-quality FRMR. The natural and environmental-friendly production of FRMR with sufficient MK is also prospected.

In the present perspective, we focus on the MLs in FRMR and their differences with lovastatin; strategies for efficient production of FRMR, the current situation of FRMR application, and the corresponding future directions for a wide application were proposed.

## Functional Substances in FRMR

Monacolins are the main bioactive substances in FRMR. MK was chemically identified as lovastatin and was first isolated from the cultures of *Monascus ruber* No. 1005 as a hypocholesterolemic agent in 1979 (Endo, 1979). MK is a polyketide compound synthesized by polyketide synthase (PKS) in *Monascus* spp. MK biosynthetic pathway and gene cluster in *Monascus* spp. are similar to those of lovastatin from *Aspergillus terreus* (Zhang et al., 2020). The MK gene cluster including nine genes named *mok A-mok I* was isolated from the genome of *Monascus pilosus*, and functions of the genes have been carried out (Chen et al., 2008; Zhang et al., 2017b). Overexpression of key genes (*mokC*, *mokD*, *mokE*, and *mokI*) in *the Monascus purpureus* azaphilone polyketide pathway can be used to improve MK production (Zhang et al., 2019).

Monacolins are chemical analogs of MK that share a similar basic skeleton, with difference in the substituent groups. MLs are mainly divided into lactone ring form and free acidic form (Li et al., 2017). At least 84 MLs have been identified, though not all of the MLs have been studied (Li et al., 2017). MLs, for instance, monacolin L, monacolin J, dihydromonacolin L, monacolin X, and compactin, etc., in both the lactone and acid forms have attracted more attention, owing to their high contents in FRMR and their well-known beneficial bioactivities (Endo and Hasumi, 1985; Endo, 1985a, b; Endo et al., 1986; Dhale et al., 2007a, b; Zhu et al., 2012; Hachem et al., 2020). With the progress in research on these compounds, more and more MLs have been isolated and characterized. The structures and functions of MLs O-S, α, β-dehydromonacolin S, 3α- hydroxy-3,5-dihydromonacolin L, 3b-hydroxy-3,5-dihydro monacolin L, α, β-hydromonacolin Q, monacolin T, monacolin U, 6a-O-methyl-4,6-dihydromonacolin L and 6a-O-ethyl-4,6- dihydromonacolin L have been explored (Li et al., 2004; Liu et al., 2013; Zhang et al., 2016, 2018a). It is interesting that an unusual aromatic monacolin analog, monacophenyl, was isolated from RMR (Liu et al., 2011). In view of this, one of the most important key points for taking full advantage of FRMR is exploring its functions and side effects.

Besides MLs, other functional substances such as pigments, ergosterol, γ-aminobutyric acid, and polysaccharides, also play a certain role in the function of FRMR (Wang et al., 2014; Liang et al., 2019). For instance, ergosterol showed remarkable lipid-lowering efficiency. Moreover, three *Monascus* azaphilone pigments of monascin, monasfluore B, and ankaflavin were discovered as ligands of lipase (Fang et al., 2017; Liang et al., 2019).

## Functions of FRMR

Functional red mold rice is widely consumed as a lipid-lowering product due to it containing MLs. Among the most commonly studied MLs, MK and its dihydro derivatives are the most active compounds for lowering lipid levels (Avula et al., 2014). However, MK exists in the inactive form naturally and undergoes reduction to its β-hydroxy acid (MKA) active form (Beltrán et al., 2019). MK in the lactone form gets absorbed from the gastrointestinal tract and gets converted into MKA in liver and non-hepatic tissues (Ertürk et al., 2003). In addition, the transformation process of MK into MKA spontaneously occurs at neutral pH, without the participation of gut microbiota. However, the lipid-lowering effect is mediated by the gut microbiota by catabolizing MKA to other compounds (Beltrán et al., 2019). *In vitro* experiments also indicated that MK could be completely converted into MKA only in alkaline solutions (Yang and Hwang, 2006).

Monacolins are usually obtained by consuming FRMR as a food supplement or from FRMR in combination with other bioactive compounds (Yang and Mousa, 2012; Heinz et al., 2016; D’Addato et al., 2017; Iskandar et al., 2020). An efficient and better tolerance in hypercholesterolemic patients was seen when FRMR was combined with other bioactive compounds. For instance, combining FRMR with guggulipid extract, chromium picolinate, berberine, and coenzyme Q_10_ showed a better tolerance and efficiency (Yang and Mousa, 2012; Di Pierro et al., 2016; Cicero et al., 2017; D’Addato et al., 2017; Stefanutti et al., 2017; Mazza et al., 2018; Formisano et al., 2019; Wang et al., 2019; Iskandar et al., 2020).

Besides the lowering lipid effects, the physiological functions of MLs like promoting bone formation, attenuating arterial thrombosis, and anticancer have also been confirmed (Wong and Rabie, 2008; Tseng et al., 2011; Tien et al., 2016; Xu et al., 2017; Wang et al., 2019). New bone formation in bone defects *in vivo* and bone cell formation *in vitro* can be stimulated and increased using RMR extract (Wong and Rabie, 2008). In addition, apoptosis on gastric cancer was induced by MLs and other components by scavenging the mitochondrial reactive oxygen species (Kurokawa et al., 2017). Monacolin X is known to attenuate the cell proliferation, migration, and ROS stress-mediated apoptosis in breast cancer cells, which provides a scope for the functional research of MLs (Nagabhishek and Madankumar, 2019). Another important function of FRMR is its strong antioxidant effect, which needs to be taken into consideration (Lee et al., 2009; Mohan-Kumari et al., 2011).

## Efficient Production of FRMR

### Optimization for Production of Monacolins

Optimization of the fermentation parameters for MK production has attracted much interest since its discovery (Tsukahara et al., 2009; Panda et al., 2010; Hp, 2012; Dikshit and Tallapragada, 2016). Liquid state fermentation (LSF) has not yielded constant results and higher production. Therefore, solid-state fermentation (SSF) is gaining an increasing popularity for multiple industrially important products such as pigments, enzymes, and antibiotics, besides MLs. SSF has been widely employed in the industrial production of FRMR, due to its advantages like maximum substrate utilization, better process control, lower chances of contamination, and easy downstream processing (Praveen and Savitha, 2012). Therefore, fermentation parameters of SSF for FRMR production, such as moisture content, fermentation temperature, and inoculum concentration have been studied extensively and discussed herewith. Generally, adjusting the moisture content to 35% (*w*/*w*) and maintaining an environmental humidity at 55∼65% is beneficial for the MK production (Subhagar et al., 2009; Feng et al., 2014). Fermentation temperature is another vital parameter for MK production. The temperature-shift cultivation is more advantageous for the MK production, when compared with the constant temperature fermentation. *Monascus* spp. are generally cultured at 30°C for their growth and at lower temperature such as 25°C or even 23°C for MK production (Tsukahara et al., 2009; Lin et al., 2017). In addition, the inoculum concentration also shows an influence on *Monascus* fermentation and MK production (Subhagar et al., 2009). Appropriate inoculum size starts the fermentation quickly and maintains the fermentation process at a good rate for metabolite production. There is a significant relation between the inoculum size and the spore concentrations of the inoculum, for instance, by adjusting them to 13% (*v*/*w*) and 10^6^ CFU/ml, respectively (Feng et al., 2014). For the speedy growth of *Monascus* spp. and avoiding contamination by other microorganisms, lactic acid and acetic acid are usually added to adjust the pH of the fermentation substrates (Xu et al., 2005; Feng et al., 2014). The methods for improving the MK production by *Monascus* strains have been screened by chemical mutagenesis or genetic engineering technology (Yang et al., 2005b; Suh et al., 2007; Wang et al., 2011). A mutant KU609 with high MK and no citrinin production has been obtained from the wild-strain *Monascus* isolate number 711 by subjecting to γ-irradiation (Suh et al., 2007). The binary vector pCAMBIA3300-*gpdA-hph-trpC* with hygromycin B phosphotransferase (*hph*) was constructed and transformed into *Monascus albidus* 9901 by *Agrobacterium tumefaciens*-mediated transformation. Two transformants H1 and H2 were selected, and the MK yields of H1 and H2 fermentation products were increased by 42.15 and 40.34%, respectively, compared with that of *Monascus albidus* 9901 (Wang et al., 2011). Moreover, the mutagenic treatment of ultrasonic wave was also employed to screen *Monascus* strain producing more MK (Yang et al., 2005b). However, the biological characteristic stability of mutants should be well studied, before commencing the industrial production of FRMR.

Besides the optimization of fermentation parameters, some novel fermentation patterns have also been employed to enhance the MK production (Panda et al., 2010; Zhang et al., 2013; Seenivasan et al., 2020). Metabolic footprinting concept has been used to improve the MK production. A strong glycolytic flux pattern was observed in the shake culture, tricarboxylic acids such as, citric acid, succinic acid, and oxalic acid, apart from glycerol and ethanol are most probably utilized for enhancing production of MK (Seenivasan et al., 2020). A co-culture of *M. purpureus* and *M. ruber* or *M. purpureus* and *Monascus kaoliang* showed positive effects on MK production (Panda et al., 2010; Suraiya et al., 2018). On the other hand, agar was tried as a carrier and the MK production of 2,047.03 mg/L was obtained, when the agar concentration, particle size, and glycerol concentration were 4%, 4 × 4 × 4 mm and 18%, respectively (Zhang et al., 2013). To meet the individual needs of consumers, novel FRMR needs to be developed.

### Novel Nutritious Substrates for FRMR Production

Novel substrates have been used for *Monascus* fermentation to enrich the types and functions of FRMR, for instance, soybean flour, finger millet, and Thai glutinous rice (Chairote et al., 2010; Venkateswaran and Vijayalakshmi, 2010; Feng et al., 2014; Table 1). Among the substrates mentioned, substrates rich in starch or protein, for example, soybean flour and *Dioscorea* are more suitable for *Monascus* fermentation and MK production (Table 1). Moreover, combining novel substrates with rice or with different grains together showed a higher MK production, than using them as sole substrate (Feng et al., 2014; Suraiya et al., 2018). However, it generally needs 2∼3 weeks of fermentation to obtain FRMR with MK content of more than 10.00 mg/g. Long-term fermentation of FRMR will increase its risk of contamination (Chairote et al., 2010; Dikshit and Tallapragada, 2016; Suraiya et al., 2018). Therefore, strategies for improving the MK production and further shortening the fermentation period need an urgent attention, especially in consideration to environmentally friendly and natural means. Improving MK production during a fixed conventional fermentation cycle, for example, 14 or 21 days, which equate to shorten the fermentation periods to obtain the required MK contents of FRMR. So, irritants have been used to improve MK production for the rapid fermentation of FRMR (Zhang et al., 2019; Zhen et al., 2019; Peng et al., 2020).

**TABLE 1:** Substrates used for FRMR production.

Substrates	Addition	Fermentation mode	Fermentation time (day)	Strain	MK (mg/g)/ detection method	References
Finger millet	Substrate	SSF	7	*M. purpureus*	0.370/HPLC	Venkateswaran and Vijayalakshmi, 2010
Adlay	Substrate	SSF	7	*M. purpureus*	1.120/HPLC	Yang et al., 2005a
*Dioscorea*	Substrate	SSF	10	*M. purpureus* NTU 301	2.584/HPLC	Lee et al., 2006
Soybean powder	40%	SSF	14	*M. pilosus* MS-1	18.733/HPLC	Feng et al., 2014
*Saccharina japonica*	Approximately 48.5%	SSF	14.49	*M. purpureus* KCCM 60168	13.980/HPLC	Suraiya et al., 2018
Mixed grains	Substrate	SSF	15	*M. pilosus* K-1140	2.310/HPLC	Pyo and Seo, 2010
Wheat bran	Approximately 25%	SSF	16	*M. sanguineus*	20.040/UV	Dikshit and Tallapragada, 2016
Millet	Substrate	SSF	20	*Monascus ruber*	7.120/HPLC	Zhang et al., 2018b
Soybean	Substrate	SSF	21	*M.* sp. K	0.892/HPLC	Hong et al., 2012
Thai rice varity *Oryza sativa* L. cv. RD6	Substrate	SSF	21	*M. purpureus* CMU001	33.790/HPLC	Chairote et al., 2010

*SSF, solid-state fermentation.*

### Improving Monacolin Production Using Irritants

For efficient production of FRMR, some nutritional and non-nutritional irritants, such as glycerol, glutamic acid, NaCl, and Chinese medicines have been used in medium or substrates, in order to improve the MK production (Lu et al., 2013; Zhang et al., 2019; Zhen et al., 2019; Peng et al., 2020) (Table 2). Generally, higher yield of MK with low cost is expected for commercial purposes. Most of the irritants mentioned above confirm to this expectation. When 10 mM glutamic acid was used in the medium, MK production increased 4.8-fold; the expressions of *mokC* and *mokG* and permeability of cell membrane were also increased (Zhang et al., 2017a, 2019). Trace of linoleic acid also achieved the likely results, which was attributed to the fact that linoleic acid increased the cyclic AMP concentration and activated protein kinase that enhanced the MK production (Huang et al., 2018).

**TABLE 2:** Irritants used for improving MK production.

Irritants	Addition	Fermentation mode	Fermentation time (day)	Strain	MK/detection method	References
Glycerol	40 g/L	LSF	7	*M. pilosus* MS-1	2.401 mg/g/HPLC (MK yield of mycelia)	Feng et al., 2015
NaCl	0.02 M	LSF	10	*M. purpureus* SKY219	Approximately 90 μg/mL/HPLC (MK yield of fermentation broth)	Zhen et al., 2019
*Dioscorea*	1%	LSF	12	*M. purpureus* NTU 568	27.9 mg/g/HPLC (MK yield of mycelia)	Lee et al., 2007
Glutamic acid	10 mM	LSF	12	*Monascus* M1	215 μg/mL/HPLC (MK yield of fermentation broth)	Zhang et al., 2017a
Citri Reticulatae Pericarpium, *Poria cocos*, and *Radix Angelicae dahuricae*	3.75% Citri Reticulatae Pericarpium, 2.55% *Poria cocos*, and 2.01% *Radix*	SSF	12	*M. ruber* M2-1	3.6 mg/g/HPLC	Peng et al., 2020
Sodium nitrate	1%	SSF	14	*M. purpureus* CCRC 31615	0.378 mg/g/HPLC	Su et al., 2003
Glutamic acid	10 mM	LSF	15	*Monascus* C8	Approximately 450 μg/mL/HPLC (MK yield of fermentation broth)	Zhang et al., 2019
Linoleic acid	512 μM	LSF	15	*M. ruber* cicc 5006	Approximately 150 μg/mL/HPLC (MK yield of fermentation broth)	Huang et al., 2018
Glycerol	26%	SSF	20	*M. purpureus* 9901	12.900 mg/g/HPLC	Lu et al., 2013
Soybean hull	Approximately 50%	LSF	30	*M. pilosus* KCCM 60084	0.02 mg/g/HPLC (MK yield of fermentation product)	Simu et al., 2018

*LSF, liquid-state fermentation, SSF, solid-state fermentation.*

MK can be enhanced by glycerol both in LSF and SSF with varying concentrations of glycerol (Lu et al., 2013; Feng et al., 2015). MK yields of fermentation broth and mycelia could be enhanced significantly, when glycerol concentration was adjusted to 6 g/L (*p* < 0.05). Concentration of MLs increased and mainly existed in the mycelia after adding glycerol, compared with that of control (Feng et al., 2015). The maximum MK yield of 2.401 mg/g in mycelia was obtained, when the glycerol concentration was 40 g/L (Feng et al., 2015). Furthermore, the maximal MK yield of 12.900 mg/g was obtained, when 26% glycerol was used in SSF, with bagasse as a carrier (Lu et al., 2013). As an environmentally friendly substance, which could be obtained from byproducts of biodiesel, the comprehensive utilization of glycerol needs to be explored in a future study (Carabajal et al., 2020).

However, most of the irritants are used in LSF instead of SSF at the present research stage. It is inferred that the low addition of the irritants can easily modulate the MK production in LSF, due to rapid mixing and quick fermentation. Irritants used in SSF needs a further study in future research.

## Strategies for Better Application of FRMR

It is well known that MK from FRMR acts as an inhibitor of cholesterol synthesis. Lovastatin and several other statins are marketed as drugs whereas FRMR is offered as a food supplement. Statins can cause side effects such as muscle damage and kidney failure, hence the side effects of FRMR need a critical consideration (Xue et al., 2017). In addition, the quantities of MK in FRMR remain widely variable (Yang and Mousa, 2012). Therefore, it is imperative to evaluate whether FRMR or MLs can be safe and efficient food supplement.

### Content Variability and Quality Standardization of FRMR

Functional red mold rice promotes the maintenance of normal blood low-density lipoprotein (LDL) cholesterol concentrations due to the presence of MLs (De Backer, 2017). FRMR containing 5∼7 mg MK is considered to be an efficient cholesterol-lowering agent equivalent to 20∼40 mg of pure lovastatin (Burke, 2015). Standardized FRMR formulation with 10 mg MLs consumed daily has shown to reduce LDL cholesterol by approximately 20% (McCarty et al., 2015). In 2011, the European Food Safety Authority (EFSA) concluded the existance of a causal relationship between the consumption of lovastatin from FRMR and “maintaining normal LDL cholesterol levels.” To obtain the claimed effect, a dose of ≥10 mg lovastatin everyday was prescribed (Efsa Panel on Dietetic Products Nutrition, and Allergies. (NDA), 2011). However, the results of percent of MK in 28 brands of RMR showed a large variability. No presence of MK was detected in two brands of RMR, and MK range in the other 26 RMR brands ranged more than 60-fold. The quantity of MK consumed per day would range more than 120-fold, compared with the recommended intake claimed by the manufacturers (Cohen et al., 2017). Some other studies indicate similar results (Heber et al., 2001; Gordon et al., 2010; Song et al., 2012). In addition, the quantity of MK in RMR supplements notified to the health authorities by the manufacturers varies by 30-fold, which is attributed to the variation in the strain and the fermentation process (De Backer, 2017). The large variation of MK content in RMR supplements could induce large difference in the lipid regulating effects within individuals, which in turn could problems to the efficiency and safety of the RMR supplements. Hence, standardization must be rigorously ensured, as in many cases, the content labeling of RMR supplement is erroneous. The MK content in RMR supplements varies due to the production of RMR with different strains and fermentation process (Patel, 2016; Dujovne, 2017). However, it is worth mentioning that MK in 0.1∼0.2% range in RMR is efficient and free of side effects (Halbert et al., 2010). Based on this, effective analytical tools such as chromatography and mass spectrometry can be used to identifiy the discrepancies. In addition, a statement on the product label is required which assures that a toxin-free, non-augmented, standardized amount of MLs would be advantageous to consumers, which will allow more predictable efficacy and better safety (Nguyen et al., 2017).

FRMR available from markets is with various MK contents, for instance, 0.4, 0.8, 1.0, 1.2, 1.5, 2.0, 2.5, and 3%. FRMR with different MK contents varies in price, and the price of FRMR is usually positively correlated with its MK content (Song et al., 2019). For instance, the price of FRMR with MK content of 2% available from the market is about US$50 per 1 kg. In addition, FRMR with different product types also varies in price^1^. Therefore, in order to meet drug quality standards, commercial lovastatin is illegally added to common RMR to imitate FRMR (Song et al., 2019). In view of this, the Hongqu Health Food Standard in 2007 (Taiwan Guardian Food No.0960406448) and the EFSA are currently reassessing the safety of a 10-mg dose of MK as a food supplement (Poli et al., 2018; Song et al., 2019). For the accuracy of MK content, many standards for MLs detection have been established. However, there is no clear requirement for inspection of MK in FRMR according to most of the standards. Some criteria clarify that the required content of MK lactone in FRMR is generally not less than 0.4%, while just a few of them mention the content requirements of MK lactone and acid forms (Poli et al., 2018; Song et al., 2019). The Standard for Chinese Medicine Yinpian Processing of Sichuan Province (2015) requires that MK lactone should serve as the quality control of FRMR and the lowest MK content should be 0.4%, which is in accordance with the standard of the “Functional red yeast rice QB/T 2847–2007.” The Standard for Chinese Medicine Yinpian Processing of Zhejiang Provine (2015) indicated that the total of MK lactone and acid in FRMR should be more than 0.3%, and the peak area of acid MK must not be less than 5% of the lactone MK peak area.

In order to distinguish commercial lovastatin from MK, some efficient detection methods such as UHPLC-QQQ-MS, UHPLC-Q-TOF-MAS, and stable isotope ration analysis (^13^C-NMR) have been employed to authenticate the FRMR (Zhu et al., 2013; Perini et al., 2017). Moreover, it is demonstrated that the analysis of δ^13^C with isotope ratio mass spectrometry could authenticate the FRMR (Song et al., 2019). All the aforementioned strategies relate to standardize the MK content in RMR and authenticate the FRMR, thereby laying the foundation for standardization of FRMR.

### Safety Evaluation of FRMR

Functional red mold rice has always been used as an alternative lipid-lowering therapy for patients who are unable to tolerate the statin therapy, due to statin-associated myalgias (Gordon and Becker, 2011). However, the variability of MK content, potential of toxic byproducts, and no clinical data on the FRMR dietary supplement indicate that the patients should be cautious before FRMR is standardized (Venhuis et al., 2016). As of date, some side effects of FRMR have been reported, such as myopathy, erectile dysfunction, and liver injury, etc. (Polsani et al., 2008; Childress et al., 2013; Mazzanti et al., 2017; Liu and Chen, 2018). On the other hand, among dyslipidemic patients with low to moderate cardiovascular risk, FRMR induces less muscle fatigue symptoms and exerts comparable lipid-lowering effects, when compared with simvastatin in single-center randomized pilot trials (Xue et al., 2017). Therefore, safety evaluation of FRMR is an urgent and important subject.

It has been confirmed that the safety profile of FRMR is similar to that of statins (Mazzanti et al., 2017). Therefore, the composition and formulation of FRMR dietary supplement is particularly important due to the presence of MLs; besides, MK may also act as HMG-CoA-reductase inhibitors (Li et al., 2004). For instance, compactin is likely to be only half as effective, with respect to HMG-CoA reductase inhibition as MK (Heber et al., 2001; Li et al., 2004). Therefore, the bioavailability of the individual MLs is difficult to determine, in the presence of MK. It may be useful to specify a total MLs content in the form of monacolin equivalents. This hypothesis suggests that FRMR can be considered an unregistered medicine (Farkouh and Baumgärtel, 2019).

In addition, citrinin is a confirmed nephrotoxic and teratogenic agent present in FRMR, which is another obstacle for using FRMR as food supplement or medicine. Therefore, *Monascus* strains with high MK production and low even undetectable citrinin have been screened (Li et al., 2020). Additives such as soybean isoflavones and NaCl were also used to reduce citrinin production (Huang et al., 2019; Zhen et al., 2019; He et al., 2020a). Meanwhile, detection of citrinin in FRMR is also a matter of great concern and HPLC is usually used to detect citrinin in FRMR (Li et al., 2020). For the efficient detection of citrinin in FRMR, immunoaffinity column is employed for citrinin extraction according to the Chinese National Standards for Determination of Citrinin in Food (GB 5009.222–2016). Moreover, additive pharmacological effects may be expected for other MLs present (Venhuis et al., 2016). It should be suggested that the consumers taking FRMR should do a blood test for cholesterol before taking the FRMR dietary supplement. It should also be noted that taking FRMR and statins at the same time can easily lead to overdosing and side effects. Without active postmarket surveillance for adverse drug reactions, the valuable signals of product safety are lost. If the current regulatory status for pharmacologically effective FRMR dietary supplements do not permit adequate warnings and active monitoring of adverse drug reactions, then their regulatory status may not be appropriate (Venhuis et al., 2016).

Based on this, some strategies like the continuous monitoring of “natural” dietary supplement safety through spontaneous reports, long-term trials, appropriate information to clinicians and consumers, and timely submission of suspect reports to regulatory agencies, should be carried out (Mazzanti et al., 2017). Moreover, three important points need to be taken into consideration: (1) Recognizing that FRMR contains a statin-like compound; (2) carefully recommending FRMR to statin-intolerant patients with a history of myositis or myopathy; (3) Documenting all alternative medicines, such as FRMR, taken by patients, in order to weigh the benefit-to-risk of co-administration of other drugs (Polsani et al., 2008). Overall, the real-world vigilance should be strengthened at different levels, including consumers, clinicians and policy-makers to promote the proper use and harmonize the regulatory status of FRMR (Raschi et al., 2018; Farkouh and Baumgärtel, 2019).

## Conclusion

Functional red mold rice has been used as a folk medicine by people suffering from hyperlipidemia. However, besides MK, other MLs, pigments, and citrinin in FRMR show multiple activities, sometimes even resulting in toxicity to the consumers. For improving the MK content and optimizing the product type of FRMR, fermentation parameters should be optimized and the used of novel substrates or irritants should be employed for FRMR production. Standardization of MK contents in FRMR and evaluation of FRMR safety should be studied in detail. Based on this, a better application of FRMR as a safe and effective lipid-lowering agent can be actualized.

## Data Availability Statement

The original contributions presented in the study are included in the article/supplementary material, further inquiries can be directed to the corresponding author.

## Author Contributions

FY drafted the main parts of the manuscript. YX contributed to parts of the manuscript. Both authors approved the manuscript.

## Conflict of Interest

The authors declare that the research was conducted in the absence of any commercial or financial relationships that could be construed as a potential conflict of interest.

## Footnotes

1 https://www.walgreens.com/store/c/red-yeast-rice/ID=361661-tier3
